# Changes in the Abundance of Grassland Species in Monocultures versus Mixtures and Their Relation to Biodiversity Effects

**DOI:** 10.1371/journal.pone.0075599

**Published:** 2013-09-30

**Authors:** Elisabeth Marquard, Bernhard Schmid, Christiane Roscher, Enrica De Luca, Karin Nadrowski, Wolfgang W. Weisser, Alexandra Weigelt

**Affiliations:** 1 UFZ – Helmholtz Centre for Environmental Research, Department of Conservation Biology, Leipzig, Germany; 2 Institute of Evolutionary Biology and Environmental Studies and Zurich-Basel Plant Science Centre, University of Zurich, Zurich, Switzerland; 3 UFZ – Helmholtz Centre for Environmental Research, Department of Community Ecology, Halle, Germany; 4 Institute of Biology, University of Leipzig, Leipzig, Germany; 5 Terrestrial Ecology/Department of Ecology and Ecosystem Management, Technische Universität München, Freising-Weihenstephan, Germany; 6 German Centre for Integrative Biodiversity Research (iDiv) Halle-Jena-Leipzig, Leipzig, Germany; Umea University, Sweden

## Abstract

Numerous studies have reported positive effects of species richness on plant community productivity. Such biodiversity effects are usually quantified by comparing the performance of plant mixtures with reference monocultures. However, several mechanisms, such as the lack of resource complementarity and facilitation or the accumulation of detrimental agents, suggest that monocultures are more likely than mixtures to deteriorate over time. Increasing biodiversity effects over time could therefore result from declining monocultures instead of reflecting increases in the functioning of mixtures. Commonly, the latter is assumed when positive trends in biodiversity effects occur. Here, we analysed the performance of 60 grassland species growing in monocultures and mixtures over 9 years in a biodiversity experiment to clarify whether their temporal biomass dynamics differed and whether a potential decline of monocultures contributed significantly to the positive net biodiversity effect observed. Surprisingly, individual species’ populations produced, on average, significantly more biomass per unit area when growing in monoculture than when growing in mixture. Over time, productivity of species decreased at a rate that was, on average, slightly more negative in monocultures than in mixtures. The mean net biodiversity effect across all mixtures was continuously positive and ranged between 64–217 g per m^2^. Short-term increases in the mean net biodiversity effect were only partly due to deteriorating monocultures and were strongly affected by particular species gaining dominance in mixtures in the respective years. We conclude that our species performed, on average, comparably in monocultures and mixtures; monoculture populations being slightly more productive than mixture populations but this trend decreased over time. This suggested that negative feedbacks had not yet affected monocultures strongly but could potentially become more evident in the future. Positive biodiversity effects on aboveground productivity were heavily driven by a small, but changing, set of species that behaved differently from the average species.

## Introduction

Numerous biodiversity experiments suggest that, all else being equal, plant communities are more productive when they contain higher numbers of species [1]. Commonly, these studies analysed differences in the performance between high- and low-diversity plant communities and in some experiments these differences were observed over several years [2], [3], [4], [5], [6], [7]. Monocultures usually provide the baseline for such studies but they have not yet been the focus of interest. However, being the reference for comparisons and analytical tools in the context of biodiversity–productivity relationships, understanding the performance of monocultures over time is of critical importance for interpreting biodiversity effects in plant communities. Long-standing agricultural knowledge suggests that a single plant species is likely to decline in its yield if grown in monoculture at the same site for multiple years [8], [9]. Obviously, plants have the potential to influence the biotic or abiotic conditions they experience. For example, plants may change the soil in which they grow for the worse by an imbalanced depletion of resources [10], the release of toxic compounds [11], [12] or the accumulation of soil-borne pathogens over time [13], [14], [15], [16], [17], [18]. Such interactions are also known as negative plant–soil feedbacks [12], [13], [19], [20], [21], [22], [23]. Similarly, host-specific foliar pathogens may accumulate in monocultures if they respond positively to host density [24]. Therefore, it is conceivable that positive plant species richness–productivity relationships are largely due to negative feedbacks in monocultures and low-diversity mixtures rather than to complementary resource-use among species in high-diversity mixtures [13], [16], [18], [23], [25], [26], [27]. On the other hand, some plant species have the potential to improve the conditions of their environment, possibly by accumulating beneficial soil biota [28], [29]. Such positive plant–soil feedbacks have mainly been studied in the context of plant invasions (see e.g. [30], [31], [32]).

Interestingly, monoculture performance over time has rarely been studied in the context of biodiversity–ecosystem functioning relationships. Some previous studies conducted in biodiversity experiments compared the performance of particular species, but this was mostly done only at a single point in time (e.g. in monoculture: [33], [34], across a diversity gradient: [33], [35], [36], [37], [38], [39], [40], [41], [42]). Where biodiversity experiments were used to compare the performance of species over multiple years the focus was usually on the biodiversity–stability relationship [6], [43], [44], [45]. To our knowledge, no study has so far explicitly addressed the question how much a potential decline of plant monocultures over time contributes to the common phenomenon of overyielding mixtures in biodiversity experiments.

Here, we present a detailed analysis of the temporal dynamics in aboveground biomass production (“productivity”) that occurred over a period of 9 years in monocultures of 60 different grassland species belonging to a large scale biodiversity experiment (Jena Experiment). Given the continuous nutrient export caused by regular mowing, a general decline of plant biomass was expected in the Jena Experiment. Therefore, we could not assess the performance of our monocultures in absolute terms. Instead, we compared the performance of our plant species in monoculture to the performance of populations of the same species within plant mixtures of the same experiment.

Specifically, we tested the following hypotheses:

1. Monocultures produce on average less aboveground community biomass than plant mixtures, due to imbalanced resource depletion and/or the accumulation of detrimental agents such as pathogens or toxins (all these mechanisms are referred to as “negative feedbacks” hereafter) and the lack of mechanisms such as complementary resource-use, facilitation or sampling in monocultures. This results in a positive net biodiversity effect.
2. On average, individual plant species’ populations produce less aboveground biomass when growing in monocultures than when growing in mixtures.
3. Over time, the productivity of individual species’ populations decreases comparatively more in monoculture than in mixture as all plots suffer from nutrient export but monocultures suffer additionally from negative feedbacks. At the species level, relative biomass change rates are therefore less positive or more negative in monocultures than in mixtures.
4. The net biodiversity effect measuring the difference between monocultures and mixtures increases over time due to a gradual augmentation of positive multi-species interactions such as complementary resource-use or facilitation in mixtures as well as negative feedbacks in monocultures that lead to their deterioration. Over time, the deterioration of monocultures becomes increasingly important for explaining positive changes in the net biodiversity effect.

## Methods

### Ethics Statement

All samples were taken on the field site of the Jena Experiment. This field site is a former arable land which the research group (represented by the University of Jena) rented from the land owner for the duration of the research grant. The land owner gave the permission to conduct this study on this site. No specific permissions were required for the field work and the data collection that the current manuscript is based on. The field studies did not involve endangered or protected species.

### Field Site and Biomass Sampling

The Jena Experiment is a grassland biodiversity experiment located in the floodplain of the river Saale near Jena, Germany (50°55′ N, 11°35′ E, 130 m above see level). The experiment was established on a former arable field with loamy soil (Eutric Fluvisol) that had received high fertilizer inputs for about four decades. In May 2002, 198 experimental plant communities containing 1, 2, 4, 8, or 16 species were sown (1000 viable seeds/m^2^). The field site had been kept fallow in the year before sowing, harrowed bimonthly, and treated with glyphosate (Roundup, Monsanto, St. Louis, Missouri, USA) in July 2001. Plot size was either 3.5×3.5 m (120 small monocultures during 2003–2007, reduced to 69 plots in 2008; i.e. two replicates per species during 2003–2007 and one replicate per species during 2008–2011; for nine of our species that were also part of another experiment not reported here, we kept the two replicates until 2011) or 20×20 m (16 large monocultures and 62 mixtures with 2–16 species). The small plots were downsized from 3.5×3.5 m to 1×1 m in 2009 and the large plots were downsized from 20×20 m to 6×6 m in 2010. The species pool composed for this experiment contained 60 common Central European grassland species typical for the regional alluvial plains. Based on a cluster analysis of ecological and morphological traits, these species had been assigned to four functional groups prior to the set-up of the experiment. According to this clustering, the species pool was composed of 16 grasses, 12 small herbs, 20 tall herbs, and 12 legumes. Species composition of each large plot was determined by a constrained random draw from the species pool. Constrains were imposed to combine the gradient in plant species richness with a gradient in the number of functional groups as orthogonally as possible. In mixtures, all species were sown with equal proportions. Plots were not fertilized but mown and weeded twice a year. The field site was divided into four blocks, to account for gradually changing characteristics of the floodplain soil. Each block contained four large plots of the species richness levels 1, 2, 4 and 8, three or four 16-species mixtures and 30 monocultures of small plot size. As such, all 60 species were present in small monoculture plots (twice until 2008) and a random subsample of 16 species (four per functional group) was present in large monoculture plots. For more details about the design, establishment and maintenance of the Jena Experiment, see Roscher et al. [46] and Fig. S1.

From 2003–2011, aboveground plant biomass was harvested on all experimental plots twice per year (in late May and in late August). For all harvests, the vegetation was clipped at 3 cm above ground in four (2003–2004, August 2005, 2006–2007), three (May 2005, 2008–2009) or two (2010–2011) randomly placed sampling frames of 0.2×0.5 m per large plot and in two (2003–2009) or one (2010–1011) randomly placed sampling frame(s) of 0.2×0.5 m per small monoculture. The harvested biomass was sorted into species and dried at 70°C for at least 48 h. Part of these data (2003–2008) and more detailed information about data collection have been published by Weigelt et al. [47].

### Data Analysis

**Figure 1:**
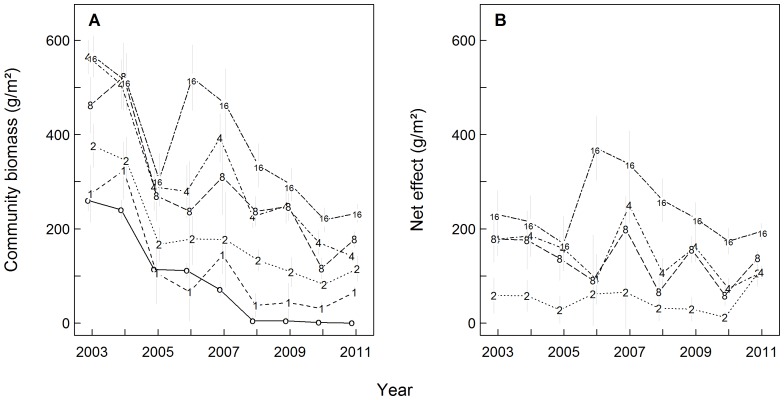
Aboveground community biomass (A) and net biodiversity effect (B) during 2003–2011. Symbols indicate medians per species richness level ±1 standard error (in A, “o” indicates monocultures of small plot size). Symbols were slightly jittered to improve visualization.

As a measure of aboveground productivity of our experimental communities and their component species we used the peak standing biomass harvested in May (averaged across all sampling frames per plot). We assessed whether, on average, positive biodiversity effects occurred and whether they increased over time by plotting the median community biomass as well as the median net biodiversity effect (see below) per species richness level over time (Fig. 1).

**Figure 2:**
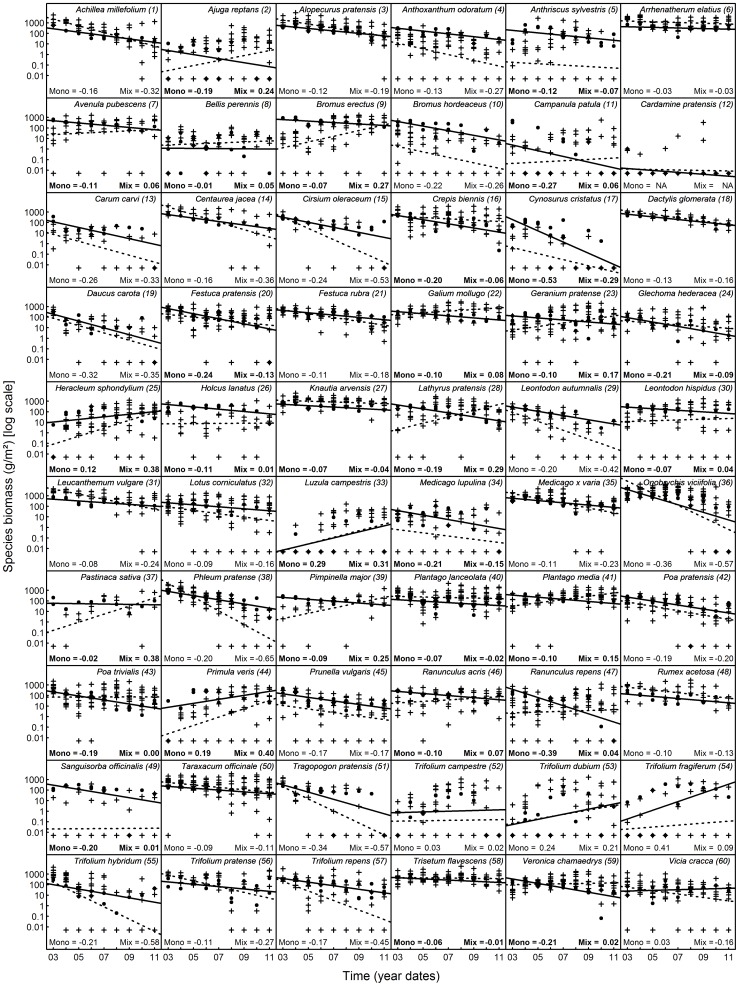
Temporal trends in species specific biomass during 2003–2011. Filled circles and solid lines indicate monocultures, crosses and broken lines indicate mixtures. Bold letters indicate less positive or more negative slopes for the regression lines across the monoculture populations than for the regression lines across the mixture populations of a particular species. Species are listed in alphabetical order; the numbers are used for their identification in Fig. 3.

To analyse differences in aboveground productivity and in temporal biomass dynamics between monocultures and mixtures at the species level we plotted the biomasses of the individual species’ populations against time (Fig. 2). For this direct comparison of yields of the individual species’ populations in monocultures and mixtures, we multiplied the species specific biomasses by the number of species that was sown into the plot on which the respective biomass was measured to correct for differences in the amounts of seeds originally used per species at the different species richness levels. A constant (0.005) was added to the resulting biomass data (corrected for sown diversity) which was then log-transformed (base 10) to improve the residual distribution.

Furthermore, we calculated relative biomass change rates (RBR) for each species in each experimental community as follows:
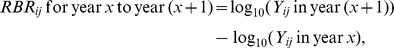
where Y_ij_ = the biomass of species i (in g/m^2^) in community j.

This metric had the advantage of being independent of sown diversity. We tested for changes in the species’ specific productivity and in RBR over time as well as for differences between monocultures and mixtures by fitting linear mixed-effects models. For each response variable (productivity and RBR) we first determined an appropriate random effects structure by comparing models with the same fixed effects, namely the monoculture-mixture contrast (MMC), the time passed since plants were sown (time), and the interaction of these two terms (MMC×time), using restricted maximum likelihood estimation. The selected random effects structure included main effects of species and harvest event (time as factor), and variable slopes for species over time. For productivity, it also included the main effect of plot as well as the interaction between MMC and time. In a second step we compared models with different fixed effects structures (built from MMC, time, and their interaction) using maximum likelihood estimation. The resulting models were compared on the basis of the Akaike information criterion, AIC, and significance was determined using likelihood ratio tests of nested models. A summary of the results of these analyses for productivity and RBR at the species level is given in Table 1. The more detailed outputs obtained by fitting the selected models to the data (including the variance components for the random terms and the estimates for the fixed terms) are given in Table S1 for productivity and in Table S2 for RBR. For productivity, the data and the model output are visualized in Fig. S2.

**Table 1:** Summary of statistical models^1^ for aboveground biomass and relative biomass change rates of individual species’ populations during 2003–2011, testing for changes over time and differences between monocultures and mixtures.

Aboveground biomass (productivity)						Biomass change rates (RBR)					
	DF^2^	AIC^2^	Chisq^2^	df^2^	Pr(>Chisq)^2^		DF	AIC	Chisq	df	Pr(>Chisq)
Nullmodel^3^	14	17337				Nullmodel	6	14208			
Time linear	15	17328	11.5	1	0.001***	Time linear	7	14210	0.3	1	0.565
MMC^4^	15	17331	8.3	1	0.004**	MMC	7	14207	3.4	1	0.066.
Time linear+MMC	16	17321	8.8	1	0.003**	Time linear+MMC	8	14209	3.4	1	0.064.
Time linear×MMC	17	17320	3.0	1	0.085.	Time linear×MMC	9	14209	1.3	1	0.263

1 Linear mixed effects models fitted by the lme4-package of the statistical software R, see Methods and Tables S1 and S2 for details. Models were fitted by stepwise inclusion of variables and p-values were inferred by their hierarchical comparison.

2 DF = model degrees of freedom, AIC = Akaike information criterion; Chisq = chi-square statistic; df = degrees of freedom required for estimating parameters, Pr(>Chisq) = associated p-value. Significance is given with *** = p<0.001;

** = p<0.01;

* = p<0.05; . = p<0.1.

3 The Nullmodel fitted an intercept, only.

4 MMC = Monoculture-Mixture-Contrast.

For every year, we calculated the net biodiversity effect for every experimental community as the difference between its observed yield (i.e., the biomass measured at the community level) and its expected yield (i.e., the average of the reference monoculture yields of the composing species, see also [48]), using the following formula:
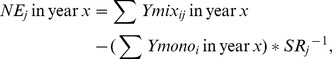
where NE_j_ = net biodiversity effect of a particular multi-species community j; Ymix_ij_ = the biomass of species i (in g/m^2^) in community j; Ymono_i_ = the biomass of species i (in g/m^2^) in its reference monoculture(s) (when two small plots were maintained for a particular species, their yields were averaged to obtain a single reference per species per year); and SR_j_ = the number of species within j. We excluded three outliers from the data set (two net biodiversity effect values from 2005 and one from 2006, as described by Marquard et al. [49]).

**Figure 3:**
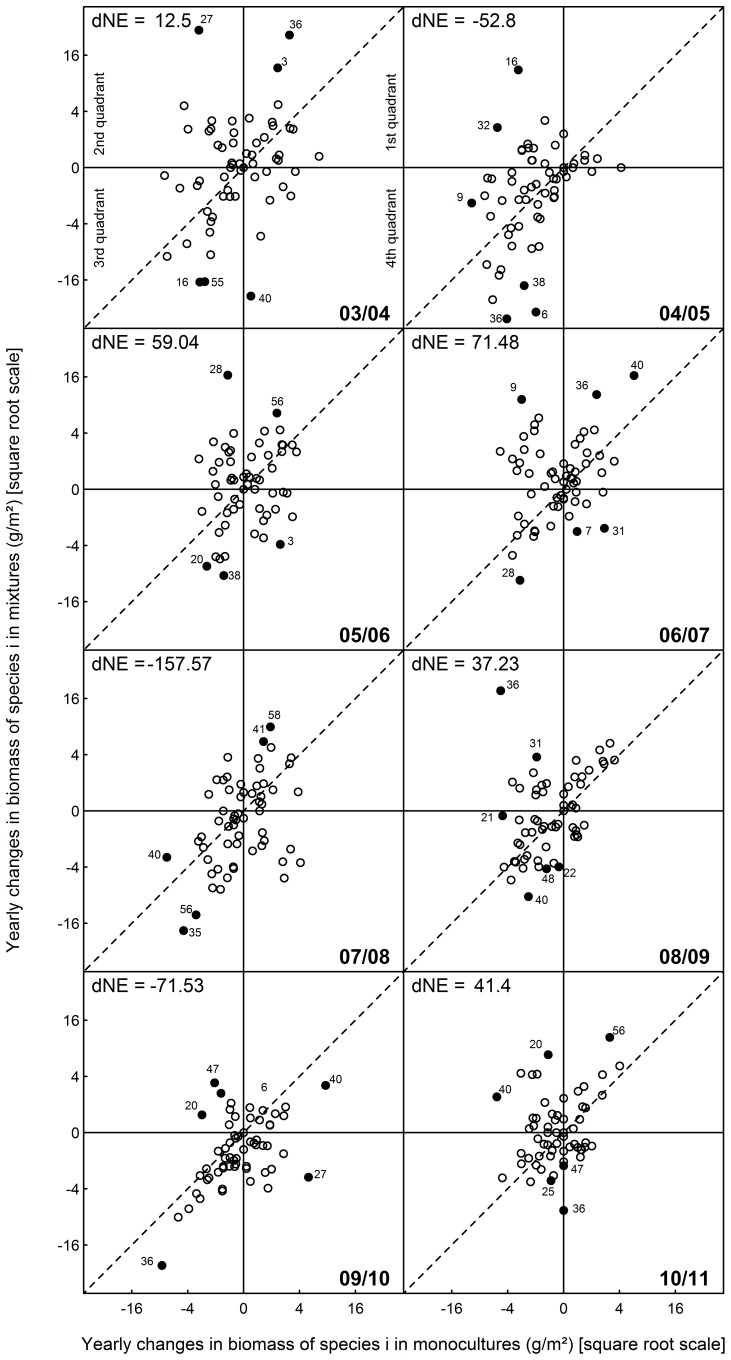
Mean yearly changes in monoculture biomass over mean yearly changes in mixture biomass per species. The panels show annual time intervals during 2003–2011 (as indicated in the lower right of each panel). The values on the x-axis equal Δ ŶE_i_ = mean changes in the expected yield of a species (monoculture yield divided by species richness); the values on the y-axis equal Δ ŶO_i_ = mean changes in the observed yield of a species (mixture yield; see Methods). A point falling on the solid vertical line indicates that a species has not changed in its expected yield (i.e., in monoculture) during the respective time interval. A point falling on the solid horizontal line indicates that a species has not changed in its observed yield (i.e., in mixture) during the respective time interval. A point falling below the broken diagonal line contributed to a decline and a point falling above the broken diagonal line contributed to an increase in the net biodiversity effect. The perpendicular distance of a point to the diagonal equals the contribution of a particular species to the change in the net biodiversity effect (see Table 2). The small numbers next to the symbols correspond to the species numbers in Fig. 2 and Table 2 and reveal the identity of the six species with the largest positive or negative contributions to changes in the net biodiversity effect. “dNE” indicates the absolute change in the net biodiversity effect (in g/m^2^) during the respective time interval. Note the square-root scale of the axes. The two most extreme values are not displayed to allow for a better scaling. These are the values for *O. viciifolia* during the time intervals 2005/2006 and 2007/2008. They are given in Table 2 (non-transformed); the contribution of *O. viciifolia* to dNE was 60.9 g/m^2^ during 2005/2006 and −106.4 g/m^2^ during 2007/2008.



To evaluate the temporal trends in the mean net biodiversity effect and whether the assumed positive changes were increasingly driven by species with deteriorating monocultures but productive populations in plant mixtures we calculated mean annual differences in the net biodiversity effect (averages across all mixtures, dNE) and analyzed how they were impacted by mean annual changes in the monoculture yield of a species (i.e., more precisely, in the expected yield of the species, Δ ŶE_i_) and mean annual changes in the mixture yield of the same species (i.e., in the terminology used here, in the observed yield of the species, Δ ŶO_i_; see Fig. 3). These metrics were calculated as follows:
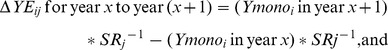


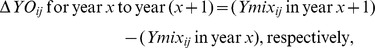
where Δ YE_ij_ = the annual difference in the expected yield of species i (in g/m^2^) in community j, Δ YO_ij_ = the annual difference in the observed yield of species i (in g/m^2^) in community j, Ymono_i_ = (the average of) the biomass of species i (in g/m^2^) in its reference monoculture(s), Ymix_ij_ = the biomass of species i (in g/m^2^) in community j, and SR_j_ = the number of species within j. For every annual time interval, we calculated the contributions of the individual species to changes in the net biodiversity effect of a particular plot (SC_ij_) as follows

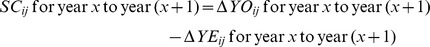



As such, we obtained positive SC_ij_-values when the annual difference in the observed yield was more positive or less negative than the annual difference in the expected yield of species i and negative SC_ij_-values when the annual difference in the observed yield was less positive or more negative than the annual difference in the expected yield of species i.

The sum of SC_ij_-values per plot j amounted to the annual change in the net biodiversity effect of the respective plant community.

For Δ YE_ij_, Δ YO_ij_ and SC_ij_, we calculated average values per species (per annual time interval: Δ ŶE_i_, Δ ŶO_i_ and ŜC_i_) and weighted them according to the proportion of plots on which species i was sown:
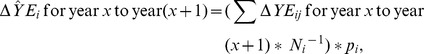


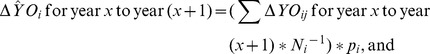


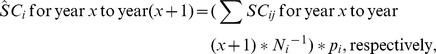
where N_i_ = the number of experimental plots containing species i, and p_i_ = the proportion of plots into which species i was sown (i.e., the number of experimental plots containing species i divided by the total number of plots). Correcting the average values per species by p_i_ was necessary to enable an analysis at the species level (instead of the plot level).

For the sake of analysing the effects of Δ ŶE_i_ and Δ ŶO_i_ on annual changes in the mean net biodiversity effect graphically, these values were square-root transformed (and the negative sign was remained if the change was negative) and two extreme data points were excluded. This allowed for a better separation of data points (see Fig. 3).

## Results

### Trends in Total Community Biomass

As a general trend, mean aboveground community biomass declined during our observation period in mixtures (from a maximum of 497 g/m^2^ in 2003 to a minimum of 141 g/m^2^ in 2010, median values computed across all multi-species plots) as well as in monocultures (from a maximum of 262 g/m^2^ in 2003 to a minimum of 0.11 g/m^2^ in 2011, median values computed across all monoculture plots; see Fig. 1A). The mean net biodiversity effect ranged from a maximum of 217 g/m^2^ in 2007 to a minimum of 64 g/m^2^ in 2010 (median values computed across all multi-species plots) and did not increase linearly over time (Fig. 1B). Aboveground biomass as well as the net biodiversity effect were positively affected by species richness (Fig. 1A and Fig. 1B; see also [49] for the period 2003–2007).

### Trends in Individual Species’ Populations

At the species level, there was considerable variation in aboveground biomass production across species, communities and years (Fig. 2 and Fig. S2). On average, the individual species’ populations produced slightly, but significantly, more (10^0.632^ = 4.3 g/m^2^) biomass when growing in monoculture than when growing in mixture (see Table 1 and Table S1: estimates for the fixed effect MMC; see also Fig. S2). Over time, the productivity of individual species’ populations in monoculture and mixture declined with marginally significantly different slopes (monoculture slope: 10^(−0.086+(−0.049))^ = 0.7, mixture slope: 10^−0.086^ = 0.8, translating into a 30% or 20% decline per year in monocultures and mixtures, respectively; see Table 1 and Table S1: estimates for the fixed effects time linear and time linear×MMC; see also Fig. S2). Consistent with these results and the biomass dynamics shown in Fig. 1 and Fig. 2, the relative biomass change rates (RBR) of the individual species’ populations were on average negative and marginally significantly different between monocultures (−0.069+(−0.083) = −0.152) and mixtures (−0.069; see Table 1 and Table S2: estimates for the intercept and MMC).

### Effects of Trends in Individual Species’ Populations on Community Net Biodiversity Effects

Positive annual changes in the mean net biodiversity effect did not predominantly or increasingly result from species proliferating in mixtures but deteriorating in monoculture (see Fig. 3, where the symbols representing the individual species are never predominantly or increasingly found in the second quadrant at the top left). Instead, we observed a large variation in biomass dynamics across species over nearly the entire study period (see the symbols scattering widely across all quadrants in most panels of Fig. 3; see also Fig. 2 and the large variance component attributed to species in Table S1). The strong decline among monocultures during 2004–2005 shown in Fig. 1 is clearly reflected in the second panel of Fig. 3 (the majority of symbols fall in the second and third quadrant). However, during that same time period, many species performed even worse in mixtures than in monocultures (see points below the broken diagonal in Fig. 3) which, overall, resulted in a decreasing mean net biodiversity effect. During all other time intervals, the large variation in species’ behaviour shown in Fig. 3 (wide scatter of symbols, relatively evenly distributed above and below the broken diagonal during most time intervals) suggested that the overall negative trends in yields shown in Fig. 1A as well as the fluctuation in the mean net biodiversity effect shown in Fig. 1B were driven by a subset of the species, only.

No species had a continuously positive or negative impact on the net biodiversity effect across the entire study period (Table 2). Nor did any of the species impact the net biodiversity effect consistently towards the direction of its overall mean (Table 2, exception: *Cardamine pratensis*, but its contributions to changes in the mean net biodiversity effect were zero during four out of eight time intervals due to very low abundances or its local extinction). Two species had particularly strong impacts on the net biodiversity effect: the perennial legume *Onobrychis viciifolia* (among the three most influential species during all time intervals) and the perennial small herb *Plantago lanceolata* (among the three most influential species in six out of eight time intervals). However, the direction of their impact was not persistent but switched between positive and negative. While the impact of *O. viciifolia* was mainly in the same direction as the mean changes in the net biodiversity effect, this was not true for *P. lanceolata* (compare Fig. 1B and Table 2). Thus, besides *O. viciifolia* and *P. lanceolata*, a varying set of species exerted strong effects on the mean net biodiversity effect. They either enhanced or weakened the mean trend in the net biodiversity effect during a particular annual time interval.

**Table 2:** Mean contributions of the individual species to mean annual changes in the net biodiversity effect (ŜC_i_) during 2003–2011.

No.	Species	ŜC_i_ 1_03/04_	ŜC_i__04/05_	ŜC_i__05/06_	ŜC_i__06/07_	ŜC_i__07/08_	ŜC_i__08/09_	ŜC_i__09/10_	ŜC_i__10/11_
*1*	*Achillea millefolium*	−3.24	−7.12	−1.19	−1.5	−1.97	1.01	−0.29	0.61
*2*	*Ajuga reptans*	0.05	−0.12	0.34	−0.04	0.58	−0.18	−0.39	0.23
*3*	*Alopecurus pratensis*	**11.09**	−8.19	**−** ***5.56***	−2.17	−4.69	−0.39	0.09	−1.24
*4*	*Anthoxanthum odoratum*	−0.51	3.4	−1.8	0.23	1.91	0.7	−0.81	0.32
*5*	*Anthriscus sylvestris*	−7.12	3.27	−1.61	6.99	−0.54	−0.81	0.1	−0.75
*6*	*Arrhenatherum elatius*	2.29	**−** ***25.4***	1.23	3.95	−3.33	0.53	**2.61**	2.32
*7*	*Avenula pubescens*	3.27	−0.39	2.67	**−** ***2.47***	0.83	0.76	−0.77	2.17
*8*	*Bellis perennis*	0.55	−0.55	−0.02	0.04	−0.11	0.2	−0.13	0.14
*9*	*Bromus erectus*	2.93	**9.26**	−1.8	**12.46**	−5.26	3.06	−3.53	6.8
*10*	*Bromus hordeaceus*	−2.45	3.19	0.32	−0.21	−0.31	2.43	−0.83	0.8
*11*	*Campanula patula*	7.92	−1.03	0.93	−0.07	0.04	−0.06	0.42	−0.43
*12*	*Cardamine pratensis*	0	0	0	0.07	−0.07	0.36	−0.36	0
*13*	*Carum carvi*	2.36	−0.12	0.3	−0.03	−0.36	−0.12	0.09	0.35
*14*	*Centaurea jacea*	1.49	−2.6	−0.37	0.83	−1.04	−0.47	−0.87	0.29
*15*	*Cirsium oleraceum*	−0.82	−0.02	0.23	−0.12	0.27	−0.17	−0.16	0.07
*16*	*Crepis biennis*	**−** ***14.08***	**14.72**	−4.78	3.26	−6.26	3.07	−3.1	−1.08
*17*	*Cynosurus cristatus*	−0.7	0.23	0.76	−0.01	0.03	0	0	0
*18*	*Dactylis glomerata*	−1.88	−2.23	1.62	2.39	−3.45	−0.96	0.09	−0.54
*19*	*Daucus carota*	−2.44	−1.37	3.73	−0.37	−1.32	0.04	−1.69	1.76
*20*	*Festuca pratensis*	4.68	3.85	**−** ***5.76***	−0.22	0.65	−3.36	**2.62**	**7.96**
*21*	*Festuca rubra*	−0.75	−1.1	−0.47	3.67	−7.8	**4.73**	−1.04	−1.12
*22*	*Galium mollugo*	1.58	2.36	−0.07	7.24	−1.67	**−** ***3.93***	0.39	−0.78
*23*	*Geranium pratense*	−1.46	1.33	−0.09	3.22	1.82	1.04	−1.36	**−** ***2.73***
*24*	*Glechoma hederacea*	0.91	0.62	0.19	−0.35	−0.21	1.33	−1.67	0.42
*25*	*Heracleum sphondylium*	−1.42	−0.38	−0.53	3.97	1.46	3.04	−3.47	1.88
*26*	*Holcus lanatus*	−3.86	2.59	−0.8	−0.12	0.43	1.28	0.92	0.06
*27*	*Knautia arvensis*	**26.48**	−15.63	−2.41	1.31	−4.09	−0.15	**−** ***7.91***	2.21
*28*	*Lathyrus pratensis*	−0.27	1.43	**16.78**	**−** ***8.06***	−2.13	1.31	−3.76	**−** ***2.22***
*29*	*Leontodon autumnalis*	−0.33	0.51	−0.35	0.19	−1.05	0.97	0.04	−0.1
*30*	*Leontodon hispidus*	1.08	1.62	1.72	0.97	0.54	1.5	−2.7	0.38
*31*	*Leucanthemum vulgare*	3.22	−9.35	−5.42	**−** ***4.06***	−7.08	**4.6**	−4.67	5.51
*32*	*Lotus corniculatus*	−3.43	**7.65**	2.78	0.07	−3.73	−0.07	−1.14	1.22
*33*	*Luzula campestris*	0	0	0	0.11	0.91	−1.11	0.34	−0.1
*34*	*Medicago lupulina*	−1.28	0.86	0.66	−1.21	−0.01	1.33	−1.42	1.03
*35*	*Medicago x varia*	3.06	6.45	−4.05	1.82	**−** ***13.57***	1.14	−1.85	5.22
*36*	*Onobrychis viciifolia*	**19.6**	**−** ***24.8***	**60.89**	**9.97**	**−** ***106.35***	**23.36**	**−** ***13.82***	**−** ***7.68***
*37*	*Pastinaca sativa*	0.22	−0.11	0.02	0.5	−0.12	−0.24	0.75	−0.31
*38*	*Phleum pratense*	9.37	**−** ***15.65***	**−** ***8.77***	−1.99	−1.34	1.38	−0.68	1.53
*39*	*Pimpinella major*	0.21	0.46	0.15	0.2	−0.02	0.71	−0.55	1.47
*40*	*Plantago lanceolata*	**−** ***20.91***	−4.96	2.12	**10.08**	**4.82**	**−** ***7.75***	**−** ***5.77***	**7.31**
*41*	*Plantago media*	1.66	6.93	3.72	2.3	**5.62**	−0.99	−1.18	0.15
*42*	*Poa pratensis*	1.11	1.27	−0.21	−0.94	1.44	−0.69	0.01	−0.12
*43*	*Poa trivialis*	−8.21	1.35	−1.1	5.56	1.97	−3.18	−1.46	−0.75
*44*	*Primula veris*	0.17	−4.24	1.24	3.35	1.74	1.46	−0.94	1.48
*45*	*Prunella vulgaris*	5.83	3.49	−2.66	−0.1	0.44	0.14	−0.38	−0.02
*46*	*Ranunculus acris*	4.09	2.64	−1.53	−1.69	0.69	0.55	1.31	−0.12
*47*	*Ranunculus repens*	−2.32	1.34	−1.76	0.53	0.11	1.01	**4.23**	−1.38
*48*	*Rumex acetosa*	0.36	−1.03	1.18	−0.97	3.21	**−** ***3.86***	−0.28	−0.48
*49*	*Sanguisorba officinalis*	−1.26	−0.84	4.06	−1.11	−0.74	3.12	−2.61	1.2
*50*	*Taraxacum officinale*	−0.8	−1.76	−3.51	1.71	4.15	−2.05	−0.82	−1.65
*51*	*Tragopogon pratensis*	−3.85	0.74	−0.27	0.46	0.31	−0.94	0.11	0.32
*52*	*Trifolium campestre*	0.11	−0.1	0.69	0.19	3.98	−2.29	−1.19	−1.08
*53*	*Trifolium dubium*	0.24	−0.01	−0.1	−1.55	0.36	1.27	−1.15	−0.3
*54*	*Trifolium fragiferum*	−0.1	−0.48	−2.02	6.37	−7.51	4.43	−0.91	0.73
*55*	*Trifolium hybridum*	**−** ***14.58***	−4.36	4.09	−0.23	−3.82	−0.05	0.21	−0.85
*56*	*Trifolium pratense*	−6.32	−6.99	**5.93**	5.44	**−** ***10.83***	0.74	−1.04	**8.77**
*57*	*Trifolium repens*	−2.23	−1.81	2.01	2.92	−5.32	3.01	−2.59	1.6
*58*	*Trisetum flavescens*	3.52	7.28	−5.25	−2.25	**7.99**	−2.51	−3.63	1.54
*59*	*Veronica chamaedrys*	0.63	2.08	2.42	0.53	2.16	−1.24	−3.1	−0.24
*60*	*Vicia cracca*	−0.96	−0.98	0.52	0.42	0.07	−0.81	0.16	−0.38

1 ŜC_i_ = mean annual contributions of the individual species to mean annual changes in the net biodiversity effect (in g/m^2^). Values were scaled according to the proportion of plots on which the species were sown (see Methods). Species are listed in alphabetical order.

2 Bold numbers indicate the three species with the highest positive contributions and numbers in bold italics indicate the three species with the most negative contributions within a particular year. These six species with the most extreme values have marked data points in Fig. 3.

## Discussion

As suggested by a wealth of studies on plant species richness–productivity relationships and in line with our first hypothesis, the monocultures of our experiment yielded on average less community biomass per unit area than the plant mixtures. Positive effects of species richness on aboveground community biomass and on the net biodiversity effect have previously been described for the Jena Experiment (see [50] for results on the May harvest in 2003; [6] for results on the May harvests from 2003–2009; and [49] for results on annual biomass data from 2003–2007). However, these publications either focused on the impact of different aspects of plant diversity on biomass production and did not study the role or the behaviour of individual species [49], [50], or analysed the role of functional turnover for the maintenance of high biomass production [6]. Here, we focused on the differences in temporal biomass dynamics between monocultures and mixtures and evaluated whether deteriorating monocultures were a considerable driver for positive biodiversity effects persisting over time.

Building upon recent findings on negative plant–soil feedbacks [13], [16], [18], [19], [26], [51], [52] we expected that species in monocultures would suffer from additional growth-limiting factors compared to those in mixtures. For example, monocultures are generally regarded as being particularly prone to deplete resources unsustainably and to accumulate detrimental agents such as pathogens or toxins over time. Therefore, plant species should produce less biomass in monocultures than in mixtures and this difference should increase with time. Interestingly, we found that individual species’ populations were on average slightly more productive when experiencing only intra-specific competition than when experiencing inter-specific competition. Furthermore, we did not find evidence for a particularly strong and consistent deterioration of monocultures over time. Instead, high and low biomasses were harvested regularly for monocultures as well as for mixture populations and, on average, the productivity of both declined at a rate that was only slightly, but marginally significantly, different (see Fig. 2 and Fig. S2). These findings contradicted our second and third hypotheses and suggest that mechanisms that disadvantage monocultures (such as negative feedbacks) were less common than expected or more variable over time. They may also have been partly balanced by positive feedbacks which were recently hypothesised to be more prominent in nature than recognised so far [23]. Furthermore, the mean net biodiversity effect did not increase steadily over time and was not predominately or increasingly driven by the deterioration of our reference monocultures (hypothesis four). Instead, the mean net biodiversity effect was always the result of a wide range of species behaviours (see Fig. 1B and Fig. 3).

However, the marginally significant differences in the slopes of the productivity curves as well as in relative biomass change rates (RBR) may still reveal ecologically significant information, given the high number of species included here and the naturally large differences between them [53]; namely that the individual species’ populations do develop slightly differently over time, possibly indicating the occurrence of negative feedbacks within the plant monocultures. On average, over the 9 years of the study, such negative feedbacks did not affect our monocultures much more strongly than the individual species’ populations were negatively affected by inter-specific competition in the multi-species environments. The small differences in the temporal dynamics observed between monoculture and mixture populations may, however, result in stronger evidence for negative feedbacks in monocultures in the long run. Generally, the temporal dynamics in aboveground productivity were very variable across species in monoculture as well as in mixture and a more focused analysis of a particular sub-set of species may deliver additional insights into the role of negative feedbacks on the development of our species. For example, we found that the difference in RBR between monocultures and mixtures was more significant (monocultures having a more negative RBR than mixtures) when we restricted our analysis to those species that were actually present in our biomass samples (i.e. to species that had biomasses >0, results not shown). This corroborated our interpretation that monoculture populations were affected by factors decreasing their performance compared to those in mixtures over time, but that these factors were not yet strong enough to detect them unequivocally across a set of 60 different grassland species.

The mean net biodiversity effect was consistently positive and clearly demonstrated an average advantage of mixtures over monocultures at the community level that was much larger than the amount to which monocultures outcompeted mixtures at the population level (64–217 g/m^2^ as compared with 4.3 g/m^2^, see Results). This apparent discrepancy suggested that at any time, a small subset of our species profited greatly from growing within a multi-species situation. A strong overyielding of these species likely overcompensated for the lower average productivity of individual species’ populations in mixture and thereby resulted in strong positive net biodiversity effects. We could only identify two species that had a strong impact on the magnitude of the net biodiversity effects during most of the years (*O. viciifolia* and *P. lanceolata*); the identity of the other influential species changed frequently over time. Such strong fluctuations in species abundance may be the result of growth rates being negatively frequency dependent [54]. Corroborating previous findings [6], [45], these results suggested a substantial turnover in the species that drive the overyielding of plant mixtures in the Jena Experiment. We conclude that these driving species were few in one context, but variable across contexts, and that different mechanisms enhanced their productivity within the mixtures, including the more efficient partitioning of resources (complementarity) and other positive inter-specific interactions (such as facilitation).

## Supporting Information

Figure S1 **Schematic view of the field site showing the location and arrangement of the plots.** Large squares symbolize the large monocultures (n = 16) and mixtures (n = 62), small squares symbolize the reference monocultures (n = 120). Small plots with grey borders were harvested until 2008. The different colour shading indicates the four blocks into which the field site was divided (see Methods for more details).
(TIF)

Figure S2 **Visualisation of model estimates for aboveground biomass data of individual species’ populations during 2003–2011.** The data (corrected for sown diversity, 0.005 added, log10-transformed) is represented by symbols (grey: mixtures, black: monocultures); the bold lines indicate the overall intercepts and slopes as determined by the fixed part of the model (broken: mixtures, solid: monocultures). The p-value relates to the difference between the slopes of the regression lines (see the time linear×MMC interaction term in Table 1).
(TIF)

Table S1 **Results of variance components analysis for aboveground biomass (productivity) of individual species’ populations during 2003–2011.**
(DOC)

Table S2 **Results of variance components analysis for species specific relative biomass change rates (RBR) during 2003–2011.**
(DOC)

## References

[1] Cardinale BJ, Matulich KL, Hooper DU, Byrnes JE, Duffy E, et al (2011) The functional role of producer diversity in ecosystems. American Journal of Botany 98: 572–592. 21613148 10.3732/ajb.1000364

[2] Fargione J, Tilman D, Dybzinski R, Hille Ris Lambers J, Clark C, et al (2007) From selection to complementarity: shifts in the causes of biodiversity-productivity relationships in a long-term biodiversity experiment. Proceedings of the Royal Society of London Series B-Biological Sciences 274: 871–876. 10.1098/rspb.2006.0351 PMC2093979 17251113

[3] Spehn EM, Hector A, Joshi J, Scherer-Lorenzen M, Schmid B, et al (2005) Ecosystem effects of biodiversity manipulations in European grasslands. Ecological Monographs 75: 37–63.

[4] Tilman D, Reich PB, Knops JM (2006) Biodiversity and ecosystem stability in a decade-long grassland experiment. Nature 441: 629–632. 16738658 10.1038/nature04742

[5] Van Ruijven J, Berendse F (2009) Long-term persistence of a positive plant diversity–productivity relationship in the absence of legumes. Oikos 118: 101–106.

[6] Allan E, Weisser W, Weigelt A, Roscher C, Fischer M, et al.. (2011) More diverse plant communities have higher functioning over time due to turnover in complementary dominant species. Proceedings of the National Academy of Sciences. 10.1073/pnas.1104015108 PMC3193239 21949392

[7] Reich PB, Tilman D, Isbell F, Mueller K, Hobbie SE, et al (2012) Impacts of Biodiversity Loss Escalate Through Time as Redundancy Fades. Science 336: 589–592. 22556253 10.1126/science.1217909

[8] Burdon JJ (1987) Diseases and plant population biology: Cambridge University Press.

[9] Mundt CC (2002) Use of multiline cultivars and cultivar mixtures for disease management. Annual Review of Phytopathology 40: 381–410. 10.1146/annurev.phyto.40.011402.113723 12147765

[10] Schenk HJ (2006) Root competition: beyond resource depletion. Journal of Ecology 94: 725–739.

[11] Singh HP, Batish DR, Kohli RK (1999) Autotoxicity: Concept, organisms, and ecological significance. Critical Reviews in Plant Sciences 18: 757–772.

[12] Bonanomi G, Rietkerk M, Dekker SC, Mazzoleni S (2008) Islands of fertility induce co-occurring negative and positive plant-soil feedbacks promoting coexistence. Plant Ecology 197: 207–218.

[13] Petermann JS, Fergus AJF, Turnbull LA, Schmid B (2008) Janzen-Connell effects are widespread and strong enough to maintain diversity in grasslands. Ecology 89: 2399–2406. 18831160 10.1890/07-2056.1

[14] Van der Putten WH, Van Dijk C, Peters BAM (1993) Plant-specific soil-borne diseases contribute to succession in foredune vegetation. Nature 362: 53–56.

[15] Diez JM, Dickie I, Edwards G, Hulme PE, Sullivan JJ, et al (2010) Negative soil feedbacks accumulate over time for non-native plant species. Ecology Letters 13: 803–809. 20482584 10.1111/j.1461-0248.2010.01474.x

[16] Schnitzer SA, Klironomos JN, HilleRisLambers J, Kinkel LL, Reich PB, et al (2011) Soil microbes drive the classic plant diversity-productivity pattern. Ecology 92: 296–303. 21618909 10.1890/10-0773.1

[17] Mordecai EA (2011) Pathogen impacts on plant communities: unifying theory, concepts, and empirical work. Ecological Monographs 81: 429–441.

[18] Maron JL, Marler M, Klironomos JN, Cleveland CC (2011) Soil fungal pathogens and the relationship between plant diversity and productivity. Ecology Letters 14: 36–41. 21073641 10.1111/j.1461-0248.2010.01547.x

[19] Casper BB, Castelli JP (2007) Evaluating plant-soil feedback together with competition in a serpentine grassland. Ecology Letters 10: 394–400. 17498138 10.1111/j.1461-0248.2007.01030.x

[20] Van der Heijden MGA, Bardgett RD, Van Straalen NM (2008) The unseen majority: soil microbes as drivers of plant diversity and productivity in terrestrial ecosystems. Ecology Letters 11: 296–310. 18047587 10.1111/j.1461-0248.2007.01139.x

[21] Bever JD, Westover KM, Antonovics J (1997) Incorporating the soil community into plant population dynamics: the utility of the feedback approach. Journal of Ecology 85: 561–573.

[22] Bartelt-Ryser J, Joshi J, Schmid B, Brandl H, Balser T (2005) Soil feedbacks of plant diversity on soil microbial communities and subsequent plant growth. Perspectives in Plant Ecology Evolution and Systematics 7: 27–49.

[23] van der Putten WH, Bardgett RD, Bever JD, Bezemer TM, Casper BB, et al (2013) Plant-soil feedbacks: the past, the present and future challenges. Journal of Ecology 101: 265–276.

[24] Mitchell CE, Tilman D, Groth JV (2002) Effects of grassland plant species diversity, abundance, and composition on foliar fungal disease. Ecology 83: 1713–1726.

[25] Mwangi PN, Schmitz M, Scherber C, Roscher C, Schumacher J, et al (2007) Niche pre-emption increases with species richness in experimental plant communities. Journal of Ecology 95: 65–78.

[26] Hendriks M, Mommer L, de Caluwe H, Smit-Tiekstra AE, van der Putten WH, et al (2013) Independent variations of plant and soil mixtures reveal soil feedback effects on plant community overyielding. Journal of Ecology 101: 287–297.

[27] Kulmatiski A, Beard KH, Heavilin J (2012) Plant-soil feedbacks provide an additional explanation for diversity-productivity relationships. Proceedings of the Royal Society B-Biological Sciences 279: 3020–3026. 10.1098/rspb.2012.0285 PMC3385476 22496190

[28] Wagg C, Jansa J, Stadler M, Schmid B, van der Heijden MGA (2011) Mycorrhizal fungal identity and diversity relaxes plant-plant competition. Ecology 92: 1303–1313. 21797158 10.1890/10-1915.1

[29] Latz E, Eisenhauer N, Rall BC, Allan E, Roscher C, et al (2012) Plant diversity improves protection against soil-borne pathogens by fostering antagonistic bacterial communities. Journal of Ecology 100: 597–604.

[30] de la Pena E, de Clercq N, Bonte D, Roiloa S, Rodriguez-Echeverria S, et al (2010) Plant-soil feedback as a mechanism of invasion by Carpobrotus edulis. Biological Invasions 12: 3637–3648.

[31] Klironomos JN (2002) Feedback with soil biota contributes to plant rarity and invasiveness in communities. Nature 417: 67–70. 11986666 10.1038/417067a

[32] Suding KN, Harpole WS, Fukami T, Kulmatiski A, MacDougall AS, et al (2013) Consequences of plant-soil feedbacks in invasion. Journal of Ecology 101: 298–308.

[33] Hector A, Bazeley-White E, Loreau M, Otway S, Schmid B (2002) Overyielding in grassland communities: testing the sampling effect hypothesis with replicated biodiversity experiments. Ecology Letters 5: 502–511.

[34] Heisse K, Roscher C, Schumacher J, Schulze ED (2007) Establishment of grassland species in monocultures: different strategies lead to success. Oecologia 152: 435–447. 17356814 10.1007/s00442-007-0666-6

[35] Dimitrakopoulos PG, Schmid B (2004) Biodiversity effects increase linearly with biotope space. Ecology Letters 7: 574–583.

[36] Hille Ris Lambers J, Harpole SW, Tilman D, Knops J, Reich PB (2004) Mechanisms responsible for the positive diversity-productivity relationship in Minnesota grasslands. Ecology Letters 7: 661–668.

[37] Roscher C, Schumacher J, Weisser WW, Schmid B, Schulze ED (2007) Detecting the role of individual species for overyielding in experimental grassland communities composed of potentially dominant species. Oecologia 154: 535–549. 17851699 10.1007/s00442-007-0846-4

[38] Troumbis AY, Dimitrakopoulos PG, Siamantziouras ASD, Memtsas D (2000) Hidden diversity and productivity patterns in mixed Mediterranean grasslands. Oikos 90: 549–559.

[39] Van Ruijven J, Berendse F (2003) Positive effects of plant species diversity on productivity in the absence of legumes. Ecology Letters 6: 170–175.

[40] Tilman D, Knops J, Wedin D, Reich P, Ritchie M, et al (1997) The influence of functional diversity and composition on ecosystem processes. Science 277: 1300–1302.

[41] Marquard E, Weigelt A, Roscher C, Gubsch M, Lipowsky A, et al (2009) Positive biodiversity-productivity relationship due to increased plant density. Journal of Ecology 97: 696–704.

[42] Roscher C, Scherer-Lorenzen M, Schumacher J, Temperton VM, Buchmann N, et al (2011) Plant resource-use characteristics as predictors for species contribution to community biomass in experimental grasslands. Perspectives in Plant Ecology Evolution and Systematics 13: 1–13.

[43] Isbell FI, Polley HW, Wilsey BJ (2009) Biodiversity, productivity and the temporal stability of productivity: patterns and processes. Ecology Letters 12: 443–451. 19379138 10.1111/j.1461-0248.2009.01299.x

[44] Van Ruijven J, Berendse F (2007) Contrasting effects of diversity on the temporal stability of plant populations. Oikos 116: 1323–1330.

[45] Roscher C, Weigelt A, Proulx R, Marquard E, Schumacher J, et al (2011) Identifying population- and community-level mechanisms of diversity-stability relationships in experimental grasslands. Journal of Ecology 99: 1460–1469.

[46] Roscher C, Schumacher J, Baade J, Wilcke W, Gleixner G, et al (2004) The role of biodiversity for element cycling and trophic interactions: an experimental approach in a grassland community. Basic and Applied Ecology 5: 107–121.

[47] Weigelt A, Marquard E, Temperton VM, Roscher C, Scherber C, et al (2010) The Jena Experiment: six years of data from a grassland biodiversity experiment. Ecology 91: 930.

[48] Loreau M, Hector A (2001) Partitioning selection and complementarity in biodiversity experiments. Nature 412: 72–76. 11452308 10.1038/35083573

[49] Marquard E, Weigelt A, Temperton VM, Roscher C, Schumacher J, et al (2009) Plant species richness and functional composition drive overyielding in a 6-year grassland experiment. Ecology 90: 3290–3302. 20120799 10.1890/09-0069.1

[50] Roscher C, Temperton VM, Scherer-Lorenzen M, Schmitz M, Schumacher J, et al (2005) Overyielding in experimental grassland communities - irrespective of species pool or spatial scale. Ecology Letters 8: 419–429.

[51] Bell T, Freckleton RP, Lewis OT (2006) Plant pathogens drive density-dependent seedling mortality in a tropical tree. Ecology Letters 9: 569–574. 16643302 10.1111/j.1461-0248.2006.00905.x

[52] Kulmatiski A, Beard KH, Stevens JR, Cobbold SM (2008) Plant-soil feedbacks: a meta-analytical review. Ecology Letters 11: 980–992. 18522641 10.1111/j.1461-0248.2008.01209.x

[53] Toft CA, Shea PJ (1983) Detecting community-wide patterns. Estimating power strengthens statistical inference. American Naturalist 122: 618–625.

[54] Chesson P (2000) Mechanisms of maintenance of species diversity. Annual Review of Ecology and Systematics 31: 343-+.

