# Supplementary figures and images for: Changes in the Abundance of Grassland Species in Monocultures versus Mixtures and Their Relation to Biodiversity Effects
Source: PLoS One. 2013 Sep 30;8(9):e75599. doi: 10.1371/journal.pone.0075599 (PMC3787038; doi:10.1371/journal.pone.0075599)

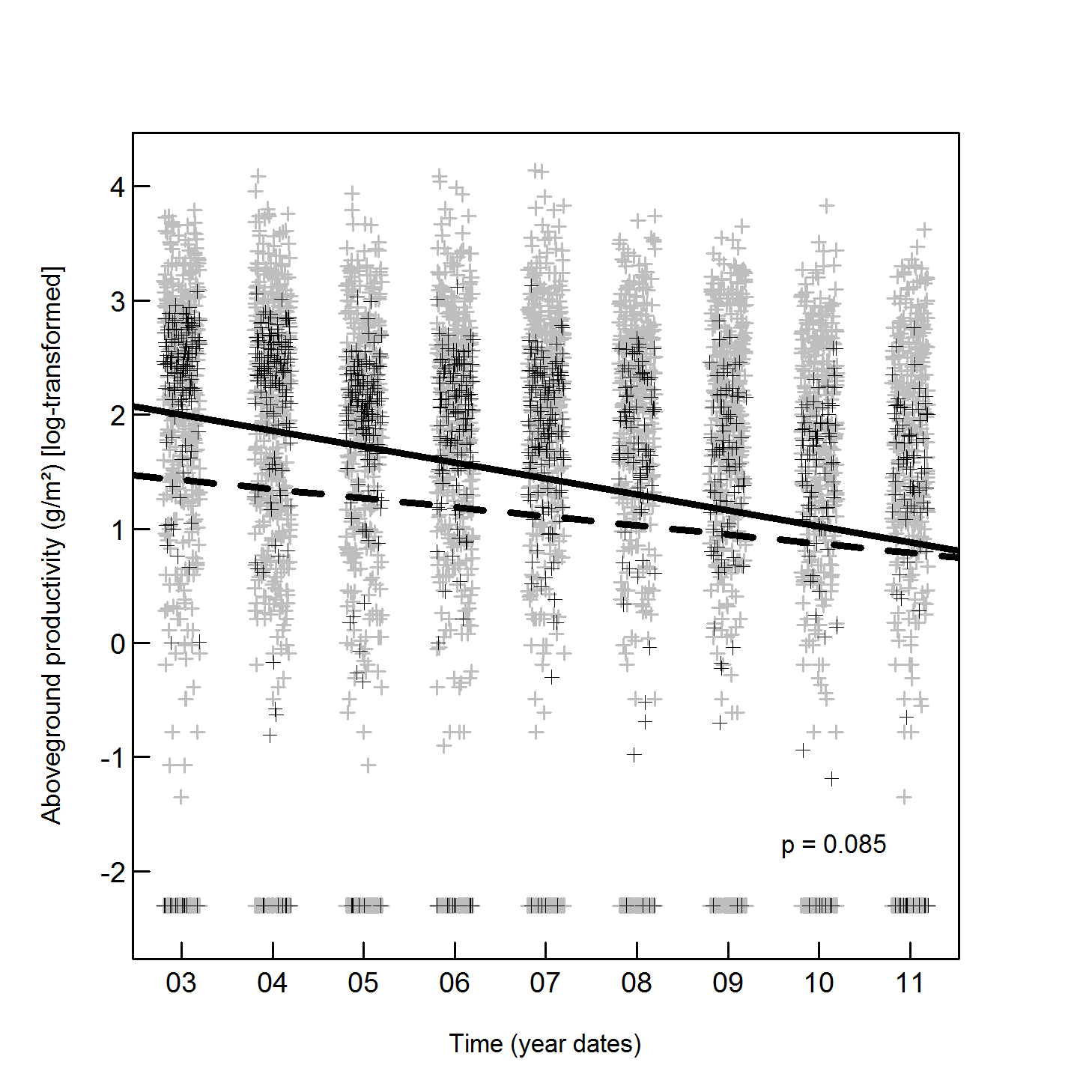

Supplement: Figure S2 — Visualisation of model estimates for aboveground biomass data of individual species’ populations during 2003–2011. The data (corrected for sown diversity, 0.005 added, log10-transformed) is represented by symbols (grey: mixtures, black: monocultures); the bold lines indicate the overall intercepts and slopes as determined by the fixed part of the model (broken: mixtures, solid: monocultures). The p-value relates to the difference between the slopes of the regression lines (see the time linear×MMC interaction term in Table 1). (TIF) [file pone.0075599.s002.tif]
