# Supplementary material for: Changes in the Abundance of Grassland Species in Monocultures versus Mixtures and Their Relation to Biodiversity Effects
Source: PLoS One. 2013 Sep 30;8(9):e75599. doi: 10.1371/journal.pone.0075599 (PMC3787038; doi:10.1371/journal.pone.0075599)
Supplement: Table S1 — Results of variance components analysis for aboveground biomass (productivity) of individual species’ populations during 2003–2011. (DOC) [file pone.0075599.s003.doc]

**Table S1.** Results of variance components analysis for aboveground biomass (productivity) of individual species' populations during 2003–2011^1^

| **Source of variation** | | | | | | | | | | | | | |
| --- | --- | --- | --- | --- | --- | --- | --- | --- | --- | --- | --- | --- | --- |
| **Random effects** | | |  | **Fixed effects** | | | | | | | |  | |
|  | Variance | Std.Dev. |  |  | Estimate | Std. Error | t-value | AIC^2^ | Chisq^2^ | df^2^ | Pr(>Chisq)^2^ | |  |
| Plot (Intercept) | 0.165 | 0.406 |  | (Intercept) | 1.517 | 0.282 | 5.38 | 17337 |  |  |  | |  |
| Species (Intercept) | 3.493 | 1.869 |  | Time linear | -0.086 | 0.039 | -2.20 | 17328 | 11.5 | 1 | < 0.001 | | *** |
| MMC^3, 4^ | 1.235 | 1.111 |  | MMC^4^ | 0.632 | 0.183 | 3.44 | 17331 | 8.3 | 1 | 0.004 | | ** |
| Time linear^3^ | 0.057 | 0.240 |  | Time linear x MMC^4^ | -0.049 | 0.028 | -1.74 | 17320 | 2.9 | 1 | 0.085 | | . |
| Time linear x MMC^3^ | 0.027 | 0.164 |  |  |  |  |  |  |  |  |  | |  |
| Time as factor (Intercept) | 0.031 | 0.176 |  |  |  |  |  |  |  |  |  | |  |
| Residual | 1.576 | 1.255 |  |  |  |  |  |  |  |  |  | |  |

^1^ Calculated by a maximum likelihood mixed effects model, using the lme4-package of the statistical software R, see Methods for details. 5044 observations; 198 plots; 60 species; 8 levels of time (the time interval 2003–2004 was encoded as 1 and the time interval 2010–2011 was encoded as 8).

^2^ AIC = Akaike information criterion; Chisq = chi-square statistic; df = degrees of freedom required for estimating parameters, Pr(>Chisq) = associated p-value. Significance is given with *** = p < 0.001; ** = p < 0.01; * = p < 0.05; . = p < 0.1.

^3^ These terms allowed variable intercepts and slopes within the species random effect.

^4^ MMC = Monoculture-Mixture-Contrast.
