# Supplementary material for: Changes in the Abundance of Grassland Species in Monocultures versus Mixtures and Their Relation to Biodiversity Effects
Source: PLoS One. 2013 Sep 30;8(9):e75599. doi: 10.1371/journal.pone.0075599 (PMC3787038; doi:10.1371/journal.pone.0075599)
Supplement: Table S2 — Results of variance components analysis for species specific relative biomass change rates (RBR) during 2003–2011. (DOC) [file pone.0075599.s004.doc]

**Table S2.** Results of variance components analysis for species specific relative biomass change rates (RBR) during 2003–2011^1^

| **Source of variation** | | | | | | | | | | | | | |
| --- | --- | --- | --- | --- | --- | --- | --- | --- | --- | --- | --- | --- | --- |
| **Random effects** | | |  | **Fixed effects** | | | | | | | |  | |
|  | Variance | Std.Dev. |  |  | Estimate | Std. Error | t-value | AIC^2^ | Chisq^2^ | df^2^ | Pr(>Chisq)^2^ | |  |
| Species (Intercept) | 0.100 | 0.316 |  | (Intercept) | -0.069 | 0.082 | -0.84 | 14208 |  |  |  | |  |
| Time linear^3^ | 0.002 | 0.047 |  | MMC^4^ | -0.083 | 0.045 | -1.84 | 14207 | 3.4 | 1 | 0.066 | | . |
| Time as factor (Intercept) | 0.048 | 0.220 |  |  |  |  |  |  |  |  |  | |  |
| Residual | 1.390 | 1.179 |  |  |  |  |  |  |  |  |  | |  |

^1^ Calculated by a maximum likelihood mixed effects model, using the lme4-package of the statistical software R, see Methods for details. 5044 observations; 198 plots; 60 species; 8 levels of time (the time interval 2003–2004 was encoded as 1 and the time interval 2010–2011 was encoded as 8).

^2^ AIC = Akaike information criterion; Chisq = chi-square statistic; df = degrees of freedom required for estimating parameters, Pr(>Chisq) = associated p-value. Significance is given with *** = p < 0.001; ** = p < 0.01; * = p < 0.05; . = p < 0.1.

^3^ This term allowed variable slopes within the species random effect.

^4^ MMC = Monoculture-Mixture-Contrast.
